# Dynamic multi-period mixed-integer non-linear programming model for equipment selection in the mining industry

**DOI:** 10.1177/25726668251348712

**Published:** 2025-06-10

**Authors:** Sena Senses, Mustafa Kumral

**Affiliations:** 1 Department of Mining and Materials Engineering, 5620 McGill University, Montreal, Canada

**Keywords:** equipment selection, fleet management, material handling systems, mixed-integer non-linear programming, operational efficiency

## Abstract

Planning an equipment fleet is a complex engineering challenge involving (1) the capacity selection of equipment pieces forming a fleet, (2) the determination of fleet size, (3) strategic timing of equipment acquisitions, and (4) ensuring compatibility between interconnected equipment. Selecting equipment type involves evaluating capacity to meet production requirements effectively while considering operational constraints. It also requires determining the optimal number of units to avoid underutilisation or redundancy. In multi-period projects, the timing of equipment acquisition further adds complexity, as decisions must align with production rates that evolve over time. Moreover, matching equipment types is essential to ensure smooth and efficient workflows. This study develops a dynamic multi-period mixed-integer non-linear programming model to optimise equipment selection, considering capital recovery, operating costs, and equipment availability under match factor and production constraints. The model's effectiveness is demonstrated through a case study encompassing greenfield and brownfield scenarios in open-pit mining operations. The greenfield scenario emphasises phased equipment acquisition to align with production ramp-up and minimising initial costs. The brownfield scenario addresses the integration of aging and new equipment to sustain operational efficiency. The results highlight the model's flexibility and applicability, offering a robust tool for optimising equipment selection while balancing cost and performance.

## Introduction

Equipment selection is a critical decision-making process that impacts the efficiency, cost-effectiveness, and profitability of operations in various industries. From transportation and logistics to construction and extractive industries, determining the optimal equipment fleet required for specific tasks is vital to meeting operational goals. The objective of equipment selection is to ensure that the adequate fleet of equipment is available to meet the required production capacity while minimising costs related to underutilisation, downtime, and overcapitalisation. In many industries, equipment selection has a direct effect on key performance indicators (KPIs) such as service levels, throughput, and operational efficiency. For example, in logistics and transportation, optimal equipment selection ensures that deliveries are made on time without overloading the system, thus minimising fuel costs and maintenance. Similarly, in construction and heavy industry, effective equipment selection balances the availability of equipment with required workload, ensuring projects are completed on time and within budget. The importance of equipment selection extends beyond simple equipment acquisition; it also influences long-term strategic decisions, including capital investments, operational scheduling, and maintenance planning. Consequently, it is considered as a core component of operational efficiency in industries that rely heavily on equipment for their operations.

Equipment selection is also crucial in extractive industries, such as mining, oil, and gas, where the challenges of remote locations, harsh working conditions, and the need for large-scale operations make equipment decisions complex and costly. Mining projects often involve handling large volumes of material, which require not only high-capacity equipment but also coordination between different pieces of machinery to ensure optimal productivity. The complexities of equipment selection are amplified by the differing requirements of greenfield and brownfield mining projects. Greenfield projects, which start without pre-existing infrastructure, require fleet planning across the entire lifecycle, from initial ramp-up to full-scale production. Ramp-up operations, particularly relevant in greenfield projects, involve a gradual increase in production from low initial levels to full capacity. Flexible equipment selection is essential to accommodate rising demands while avoiding overcommitment of resources in early phases. Effective ramp-up strategies mitigate operational risks, ensure smooth transitions, and establish a strong foundation for long-term success. The challenge lies in achieving adequate fleet size to meet production targets while avoiding overinvestment during the early stages. In contrast, brownfield projects focus on optimising and modernising existing equipment to meet expanded production goals, often through fleet upgrades or replacements. Both scenarios demand careful alignment of equipment capacity with projected demand, factoring in economic and operational constraints. In open-pit mining, trucks and shovels are widely preferred as the primary material handling system due to their flexibility, high mobility, short deployment times, and comparatively low infrastructure requirements. These fleets generally consist of 10–70 trucks, 2–15 shovels, and 5–10 auxiliary units, which work together to accomplish key tasks such as loading, hauling, dumping, and providing support services.

**Figure 1.**
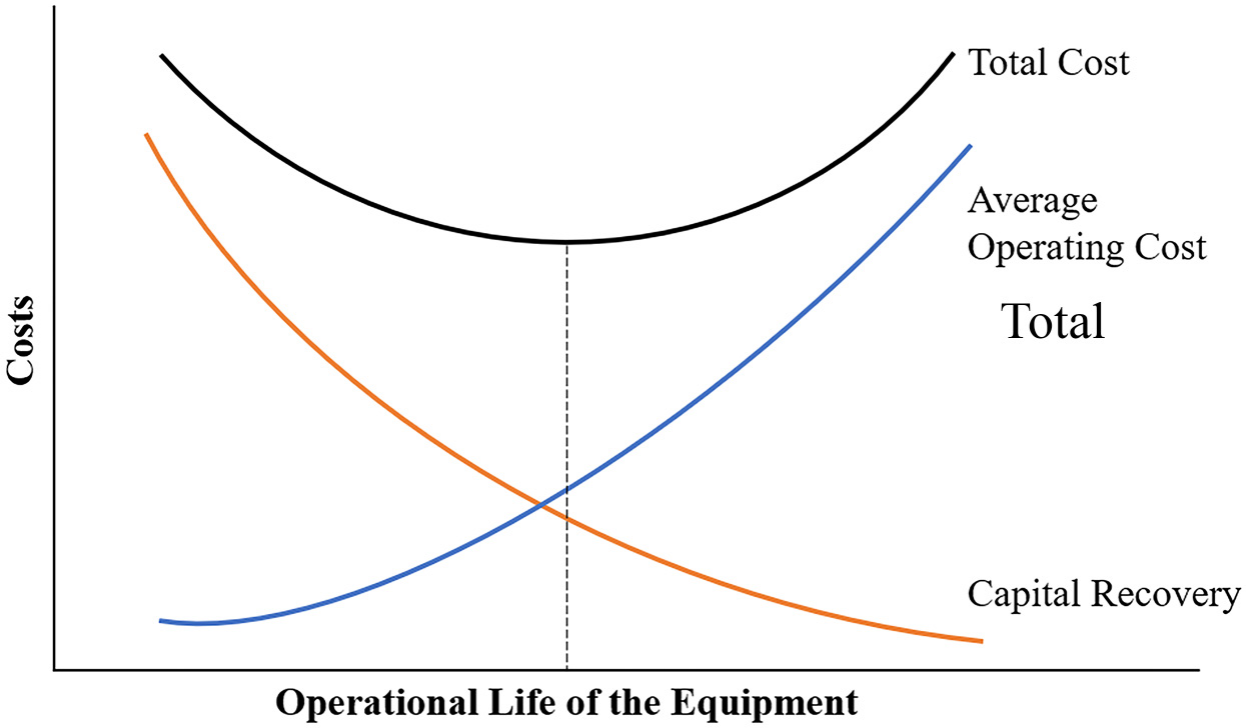
Relationship between operating and capital recovery costs.

In the mining industry, equipment selection is a multifaceted process that involves not only optimising the capacity and number of machines but also ensuring that they are available at the right time with the appropriate capacity and are deployed efficiently. In other words, this process considers several key factors, including capital cost, operational costs, equipment matching, and the strategic timing of acquisitions. The capital cost is a significant consideration in equipment selection because it directly affects the financial feasibility of a mining operation. Given that the lifespan of a mining operation often exceeds the operational life of individual pieces of equipment, equipment selection must account for multiple equipment replacement cycles over the project duration. This underscores the importance of capital cost not merely as an initial expenditure but as a recurring investment. In this context, capital recovery involves systematic allocation and distribution of the initial investment in equipment over its operational lifespan, which is achieved through depreciation schedules. Effective capital recovery ensures that the financial burden of equipment acquisition is mitigated over time. It also facilitates the planning of future equipment replacements by integrating depreciation and anticipated cash flow into the broader economic framework of the mining project. On the other hand, operational costs include all the ongoing expenses associated with running a mining fleet, such as fuel, maintenance, labour, and spare parts replacement. The loading and hauling stages in open-pit mining are among the most cost-intensive aspects of operation, typically accounting for 50% of total operating costs, underscoring the need for efficient load-and-haul systems (Huayanca et al., 2023). Figure 1 demonstrates the inverse relationship between capital recovery cost and the average operating cost of equipment over years. As the operational life increases, average operating costs tend to rise due to aging and maintenance needs, while capital recovery costs decline as the equipment's initial investment is amortised over a longer period.

Given the substantial financial burden, effective equipment selection has become an essential strategy for cost optimisation. When trucks and shovels are selected and sized to match site conditions, haulage systems not only reduce operating costs but also improve overall economic performance (Mohtasham et al., 2022). Building on cost considerations, effective equipment selection involves deeper analysis to ensure operational efficiency and cost-effectiveness. It involves determining not only the number of equipment units but also their capacities to ensure the fleet is sufficiently equipped to achieve production targets without resulting in underutilisation or inefficiencies. At this point, the timing of equipment acquisition further adds complexity, as equipment acquisitions must be strategically scheduled across multiple periods to align with anticipated changes in production volumes, equipment lifecycles, and market conditions.

In addition, equipment matching ensures seamless coordination between equipment types to optimise productivity. A fundamental concept in this regard is the match factor, a metric representing the balance between shovel and truck outputs. Originally introduced by Caterpillar, the match factor concept ensures efficiency in the loading and hauling cycle by balancing truck arrivals with loader service rates, accounting for both loading and cycle times (Burt and Caccetta, 2007; Mohtasham et al., 2021). It focuses on determining the ideal haulage fleet size, as it helps eliminate idle time by aligning equipment operation. A match factor of one represents a well-coordinated system, with trucks and loaders working at peak efficiency and no downtime. When the match factor is less than one, loaders experience idle periods due to an insufficient number of trucks to keep operations continuous. In contrast, a match factor greater than one indicates an excess of trucks, leading to delays as trucks wait for loaders to become available (Mohtasham et al., 2021).

Considering all these key factors, selecting the most appropriate equipment fleet poses a considerable challenge for decision-makers due to the extensive range of commercial options available and the complex array of criteria that must be evaluated. Decision-makers must consider factors such as equipment compatibility, operational efficiency, cost implications, and adaptability ramp-up operations in general to varying operational requirements (Oukil, 2024). Thus, a systematic approach to equipment selection is essential to ensure alignment with immediate operational requirements and long-term strategic goals, supporting an organisation's success, growth, and competitiveness.

## Background

### Multi-criteria decision-making (MCDM) approaches

Traditionally, equipment selection has been heavily reliant on experience and expert judgement. This reliance is evident in the extensive use of MCDM methods in the literature, which often incorporate expert opinions to evaluate and prioritise alternatives. A variety of fuzzy-based models have been introduced to address the uncertainty and complexity inherent in equipment selection. Baykasoǧlu et al. (2013) applied a novel fuzzy multiple attribute decision-making (MADM) model integrating fuzzy Decision-Making Trial and Evaluation Laboratory and fuzzy hierarchical TOPSIS (Technique for Order of Preference by Similarity to an Ideal Solution), providing an effective solution to the truck selection problem. Yazdani-Chamzini (2014) introduced a model combining fuzzy AHP (analytic hierarchy process) and fuzzy TOPSIS, while Nguyen et al. (2016) proposed an integrated MCDM model combining fuzzy AHP and fuzzy ARAS (additive ratio assessment), for material handling equipment selection. The fuzzy TOPSIS method (Yavuz, 2016) and an integrated AHP and fuzzy weighted-sum model methodology (Malli et al., 2021) are utilised to address truck selection in an open-pit mine. Ulutaş et al. (2023) developed a fuzzy MCDM model for forklift selection, employing fuzzy best–worst method and fuzzy multiple criteria ranking by alternative trace. Oukil (2024) presented a model based on the market weight scheme, for material handling equipment selection. Čelebić et al. (2024) developed an integrated fuzzy logic and fuzzy AHP model to select dump trucks.

Beyond fuzzy approaches, a range of MCDM techniques have been adopted. Bodziony et al. (2016) employed the ELECTRE III (Elimination and Choice Expressing the Reality) method within a multiple criteria decision aiding, while Chakraborty and Prasad (2016) developed a QFD-based expert system, and Patyk and Bodziony (2018) introduced a methodology using the APEKS to select haul trucks. Zaerpour et al. (2019) developed an ExcelVR-based decision support system to optimise storage system selection, while Ulutaş (2020) employed an integrated MCDM model combining preference selection index and weighted Euclidean distance-based approach to select stackers. Moreover, Goswami and Behera (2021) developed an integrated MCDM system using entropy and ARAS and complex proportional assessment methods, while Horňáková et al. (2021) applied the AHP to select optimal material handling equipment. Patyk and Bodziony (2022) proposed an AHP-based MCDM methodology for selecting equipment to exploit secondary deposits in surface mining operations. Shehadeh et al. (2022) introduced a multi-objective optimisation model using a genetic algorithm to determine the optimal selection and quantity of trucks and shovels for construction projects. Rakhmangulov et al. (2024) proposed a universal system of criteria for selecting dump trucks in open-pit mining, addressing multiple factors and ranking them using full consistency method.

### Simulation-based approaches

Simulation-based approaches have also been widely employed to model complex systems and assess equipment selection under various scenarios. Tan et al. (2012) constructed a simulation model utilising GPS tracking data to assess the optimal fleet composition for an open-pit copper mine. Ortiz et al. (2014) applied a discrete-event simulation (DES) model using the Arena software to evaluate fleet selection for an iron ore mining project. Dindarloo et al. (2015) developed a DES model to support equipment selection for open-pit mining, focusing on the stochastic nature of loading and haulage processes. Tiile et al. (2016) employed a DES model to identify the optimal truck–shovel combination for achieving daily mining targets in an open-pit mine. Chaowasakoo et al. (2017) addressed the truck and shovel fleet pairing problem in open-pit mining by evaluating the impact of match factors across three fleet configurations: a heterogeneous shovel fleet, a heterogeneous truck fleet, and a heterogeneous truck and shovel fleet. Moradi-Afrapoli et al. (2019) developed a multi-objective simulation–optimisation framework, incorporating match factor to optimise fleet requirements in open-pit mining. The model demonstrated a 16% reduction in fleet size compared with deterministic methods. Mustaffa (2022) employed Monte Carlo simulation to assess the impact of alternative earthmoving techniques on emissions and production efficiency in truck and shovel operations. Huayanca et al. (2023) proposed a stochastic methodology using DES model to support the replication of a yearly production plan for an open-pit copper mine, preparing for operational expansion.

### Optimisation and operational research approaches

Operational research techniques have emerged as a robust alternative, providing structured models that support the optimisation of equipment selection to achieve cost minimisation and operational efficiency. Duran and Praščević (2021) investigated the selection and allocation of construction equipment for multiple projects within a defined budget. A fuzzy linear programming approach, incorporating fuzzy objective functions and constraints, was utilised to ensure timely project completion while meeting budgetary requirements. Yang et al. (2022) utilised a multiple-objective hybrid particle swarm optimisation method for a reconfigurable assembly system to address scheduling and equipment selection problems in industrial operations. Akulker and Aydin (2023) introduced a multi-period mixed-integer non-linear programming (MINLP) model for optimal equipment selection and scheduling in the design of multi-energy microgrids. Zhao et al. (2024) presented a multi-objective optimisation model for selecting the type and determining the quantity of logistics equipment in intelligent distribution warehouses.

In the mining industry, these operational research techniques are tailored to address unique challenges, such as managing heterogeneous fleets, ensuring equipment matching, and meeting production targets under varying operational conditions. Burt et al. (2005) presented a mixed-integer linear programming model to optimise equipment selection to minimise materials handling costs for trucks and loaders. While the model demonstrated that heterogeneous fleets could lead to significant cost savings, it focused solely on single-period cost minimisation, without accounting for multi-period planning, match factor constraints, or the integration of capital and operating costs over time. Krause and Musingwini (2007) evaluated truck fleet size estimation in open-pit truck–shovel systems by modifying the machine repair model and validating it through a case study at an operating open-pit coal mine. Fu et al. (2014) developed a mixed-integer programming model to optimise the truck fleet schedule in surface mining by minimising maintenance costs. Incorporating a multi-year planning horizon and the option for new truck purchases, the model provides a flexible and cost-effective approach to managing heterogeneous truck fleets. The model's reliability was demonstrated through a case study using maintenance cost data from a gold mine, effectively reducing maintenance expenses while optimising fleet scheduling. While effective in reducing maintenance expenses, the model did not incorporate match factor constraints for heterogeneous truck and shovel combinations or account for capital and operating costs throughout the planning horizon. Mohtasham et al. (2021) developed two MINLP models to address the equipment selection problem in open-pit mining, focusing on optimising fleet efficiency by minimising deviations from target match factor values. In the first approach, suitable truck types were selected for each loader individually, while in the second, trucks were assigned to multiple loaders simultaneously. The model offered valuable insights into optimising fleet efficiency in open-pit mining but did not incorporate capital or operational cost considerations, which are crucial for a comprehensive evaluation of long-term cost-effectiveness. Hazrathosseini and Moradi Afrapoli (2024) presented a critical review on the transition towards intelligent fleet management systems in open-pit mines. They introduced a five-feature-class scale encompassing production, shovel, truck, operation, and destinations to compare existing studies and identify neglected or underexplored aspects. The authors highlighted key challenges, including multi-agent coordination and dynamic environment modelling, and proposed directions for more robust fleet management solutions under Mining 4.0 principles.

In this study, a MINLP model is developed to optimise multi-period equipment selection problem, addressing the balance between operational efficiency and cost minimisation. The main contributions of this study are as follows: (1) the adoption of a multi-period optimisation framework that enables dynamic fleet size management, accommodating changes in operational requirements, equipment performance, and costs over time; (2) a detailed cost assessment framework that incorporates both capital recovery and operating costs across multiple periods, facilitating a comprehensive financial evaluation for the equipment selection; (3) the integration of match factor constraints alongside equipment compatibility considerations between shovels and trucks to enhance fleet alignment and operational efficiency within a heterogeneous fleet; and (4) the inclusion of availability metrics based on equipment engine hours, supporting a robust, multi-period optimisation approach that accurately reflects the constraints and variability of real-world operation conditions. The model's effectiveness is demonstrated through a case study of two distinct open-pit mining scenarios: a greenfield project requiring a completely new equipment fleet and a brownfield project leveraging existing operational equipment. This application highlights the model's practical applicability and its potential to yield significant improvements in cost efficiency for operations.

## Methodology

### Equipment availability

Availability is a critical metric used to evaluate the performance of repairable systems, representing the probability that a system is operational at any given time. The analysis of availability is based on the assumption that the system alternates between two states, operating and down, forming an alternating renewal process. The times spent in these states are modelled as independent and identically distributed random variables, with periods of operation and repair following exponential distributions. These assumptions form the basis of a Markovian model, characterised by its memoryless property, where the future state of the system depends only on its current state. The failure rate ( λ ) is defined as the reciprocal of the mean time to failure (MTTF), such that $\lambda =\frac{1}{MTTF}$ , and the repair rate ( μ ) is the reciprocal of the mean time to repair (MTTR), such that $\mu =\frac{1}{MTTR}$ . The steady-state availability, which represents the long-term fraction of time the system remains operational (Tobias and Trindade, 2011), is given by as follows:

(1) $$A=\frac{\mu }{\mu +\lambda }=\frac{MTTF}{MTTR+MTTF}.$$

Instantaneous availability, on the other hand, captures the system's probability of being operational at any specific time *t*, which is particularly useful when analysing short-term performance or when the system has just begun operation. For a system operating under the assumptions outlined above, the instantaneous availability (Pham, 2003) is expressed as follows:

(2) $$A(t)=\frac{\mu }{\mu +\lambda }+\frac{\lambda }{\mu +\lambda }e^{-(\mu +\lambda )t}.$$

In this expression, the first term corresponds to the steady-state availability, while the second term represents the transient component that decreases exponentially over time. In this study, equipment availability is analysed on a multi-period basis to capture yearly variations, enabling a more realistic assessment of fleet performance throughout the project lifecycle.

### Equipment match factor

The concept of match factors, originally developed by Burt and Caccetta (2007; 2008), has been extended to address various operational scenarios involving truck–shovel systems. These extensions enhance the accuracy of the match factor calculation and are tailored for three specific situations: fleets with heterogeneous trucks, fleets with heterogeneous shovels, and fleets with both heterogeneous trucks and shovels. For fleets consisting of multiple types of trucks operating with a single type of shovel, the match factor is computed as follows:

(3) $$MF=\frac{nt\cdot \sum_{j}\;(tl_{j})}{ns{\cdot \sum }_{j}\;(tc_{j})},$$

where nt and ns represent the total number of trucks and shovels, respectively. The parameter $tl_{j}$ represents the time required to load truck *j* using the given shovel type, and $tc_{j}$ denotes the cycle time of truck *j*.

When dealing with fleets that involve multiple types of shovels but a single truck type, the match factor is determined using the following:

(4) $$MF=\frac{nt\cdot lcm(ult)}{ns\cdot \sum_{i}\;\left(\frac{lcm(ult)}{ult_{i}}\right)\cdot tc}.$$

In this case, lcm(ult) is the least common multiple of the unique loading times ( $ult_{i}$ ) across all shovel types, where $ult_{i}$ is the loading time for shovel *i* and tc represents the average cycle time of the trucks.

For fleets that involve both heterogeneous trucks and heterogeneous shovels, the match factor is calculated using the following expression:

(5) $$MF=\frac{nt\cdot \sum_{i}\;\sum_{j}\;(lt_{i,j})}{ns\cdot \sum_{j}\;(tc_{j})}.$$

where $lt_{i,j}$ represents the loading time of shovel *i* when paired with truck j *.* In this study, match factor calculation is specifically applied to evaluate the operational efficiency of heterogeneous truck–shovel fleets, ensuring optimal equipment combinations across both greenfield and brownfield scenarios.

### Capital cost recovery

The capital recovery factor (CRF) is a widely used concept for determining the equivalent uniform annual cost of an initial investment over its useful life, considering the time value of money. By standardising the recovery of an initial capital cost, the CRF enables the determination of a fixed annual recovery amount ( A ) from an initial investment ( P ). This calculation incorporates the discount rate ( r ) and the equipment's useful life in years ( n ), ensuring that both depreciation and the cost of capital are considered. The CRF is derived using the following formula (Leland Blank and Anthony Tarquin, 2012):

(6) $$CRF=\frac{r{\left(1+r\right)}^{n}}{{\left(1+r\right)}^{n}-1},$$

which represents the factor used to distribute the initial investment evenly over its useful life. Using this factor, the equivalent annual recovery cost is expressed as:

(7) $$A_{CRF}=P\cdot CRF.$$

This approach provides a reliable method for evaluating long-term investments by ensuring that the financial burden of the initial capital cost is spread uniformly across its operational life. While the CRF focuses on incorporating the time value of money, the straight-line depreciation method provides a simpler approach for allocating costs over an asset's lifespan. This method assumes a uniform decrease in the value of an asset over its useful life, distributing the capital cost evenly across each year. The annual depreciation (Leland Blank and Anthony Tarquin, 2012) is calculated as follows:

(8) $$A_{SL}=\frac{P-S}{n},$$

where *P* is the initial cost, *S* is the salvage value, and *n* is the useful life in years. This method is straightforward and widely accepted, making it suitable for financial evaluations where consistent depreciation is adequate.

The declining balance depreciation method accelerates the depreciation of an asset by applying a fixed percentage ( d ) to the book value at the beginning of each year. This approach assumes a higher depreciation expense in the early years of an asset's life, with the annual depreciation amount decreasing over time. If the fixed percentage is not explicitly stated, it can be calculated (Leland Blank and Anthony Tarquin, 2012) using the salvage value ( S ) and the initial cost ( P ) of the asset as follows:

(9) $$d=1-{\left(\frac{S}{P}\right)}^{\left(1/n\right)},$$

where *n* represents the useful life of the asset. The depreciation for a specific year *t* is then calculated using the formula:

(10) $$A_{DB}(t)=dP(1-d{)}^{\left(t-1\right)}.$$

In contrast to the other two capital recovery methods, the declining balance approach accounts for the accelerated loss of value in the early years of an asset's life. This characteristic makes it particularly well-suited for industries like mining, where equipment undergoes rapid depreciation due to intensive use, providing a more realistic alignment of costs with the asset's diminishing value over time. In this study, declining balance method is employed to calculate capital recovery, providing a systematic approach to assess the depreciation of equipment investments across different project phases.

### MINLP model development

#### Heterogeneous Equipment Selection Model without existing equipment

The Heterogeneous Equipment Selection Model is designed to optimise the number and size of equipment fleets in operations involving multiple types of trucks and shovels with varying capacities, costs, and performance characteristics. The model accounts for the complex interplay between equipment heterogeneity, operational constraints, and minimum annual production requirements, assuming that the project is initiated with a newly acquired fleet. By integrating key factors such as availability, match factor, and capital recovery, the model provides a comprehensive framework for fleet optimisation that balances operational efficiency and economic feasibility. The sets, parameters, and variables utilised in the model are presented as follows.

**Table:** 

Set	
I	Shovel types
J	Truck types
T	Time in years
S	Set of incompatible truck and shovel combinations (i,j)
Parameters	
w	Total working hours in a year
$PR_{t}$	Minimum annual production requirement in year t
$C_{i,t}^{c,\delta }$	Capital recovery of shovel type *i* in year t
$C_{\;j,t}^{c,\zeta }$	Capital recovery of truck type *j* in year t
$C_{i,t}^{o,\delta }$	Operating cost of shovel type *i* in year t
$C_{\;j,t}^{o,\zeta }$	Operating cost of truck type *j* in year t
$L_{j}$	Capacity of truck type j
$A_{\;j,t}$	Availability of truck type *j* in year t
$LT_{i,j}$	Loading time of shovel type *i* and truck type j
$CT_{\;j,t}$	Average cycle time of truck type *j* in year t
$NTR_{i,j,t}$	Number of trips made by truck type *j* for shovel type *i* in year t
$MF_{min}$	Minimum required match factor of the fleet
$MF_{max}$	Maximum required match factor of the fleet
M	Large constant
Decision Variable	
$NL_{i,t}*$	Number of new shovels of type *i* purchased in year t
$NT_{i,j,t}*$	Number of new trucks of type *j* for shovel type *i* purchased in year t
$NL_{i,t}$	Total number of shovels of type *i* operating in year t
$NT_{i,j,t}$	Total number of trucks of type *j* for shovel type *i* operating in year t
$MF_{t}$	Match factor in year t
$P_{t}$	Total production in year t

The general expression of MINLP model is shown below.

**Table:** 

$Min\;\underset{t\in T}{\sum }\;f_{1}(t)+f_{2}(t)$		(11)
$f_{1}(t)=\underset{i\in I}{\sum }\;\underset{t_{2}\in T,\;t\geq t_{2}}{\sum }\;(C_{i,(t-t_{2}+1)}^{c,\delta }+C_{i,(t-t_{2}+1)}^{o,\delta })\cdot NL_{i,t_{2}}*\cdot w$	∀t∈T	
$f_{2}(t)=\underset{i\in I}{\sum }\;\underset{\;j\in J}{\sum }\;\underset{t_{2}\in T,\;t\geq t_{2}}{\sum }\;(C_{\;j,(t-t_{2}+1)}^{c,\zeta }+C_{\;j,(t-t_{2}+1)}^{o,\zeta })\cdot NT_{i,j,t_{2}}*\cdot w$	∀t∈T	
*Subject to*		
$NL_{i,t}=NL_{i,t}*$	∀i∈I,t=1	(12)
$NL_{i,t}=NL_{i,t-1}+NL_{i,t}*$	∀i∈I,t>1	(13)
$NT_{i,j,t}=NT_{i,j,t}*$	∀i∈I,j∈J,t=1	(14)
$NT_{i,j,t}=NT_{i,j,t-1}+NT_{i,j,t}*$	∀i∈I,j∈J,t>1	(15)
$NL_{i,t}\leq M\cdot \underset{\;j\in J}{\sum }\;NT_{i,j,t}\;$	∀i∈I,t∈T	(16)
$\underset{\;j\in J}{\sum }\;NT_{i,j,t}\;\leq NL_{i,t}\cdot M$	∀i∈I,t∈T	(17)
$MF_{t}=\frac{\sum_{i}\;\sum_{j}\;NT_{i,j,t}\;\;}{\sum_{i}\;\left(\frac{NL_{i,t}}{\sum_{j}\;(NT_{i,j,t}\cdot LT_{i,j}\;)}\right)\cdot \sum_{i}\;\sum_{j}\;(NT_{i,j,t}\cdot CT_{\;j,t})\;}$	∀t∈T	(18)
$MF_{t}\geq MF_{min}$	∀t∈T	(19)
$MF_{t}\leq MF_{max}$	∀t∈T	(20)
$P_{t}=\underset{i\in I}{\sum }\;\underset{\;j\in J}{\sum }\;\underset{t_{2}\in T,\;t\geq t_{2}}{\sum }\;NT_{i,j,t_{2}}*\cdot NTR_{i,j,t}\cdot L_{j}\cdot A_{\;j,(t-t_{2}+1)})\;$	∀t∈T	(21)
$P_{t}\geq PR_{t}$	∀t∈T	(22)
$NT_{i,j,t}=0$	∀(i,j)∈S,t∈T	(23)
$MF_{t},P_{t}\;\geq 0$	∀i∈I,j∈J	(24)
$NL_{i,t},NL_{i,t}*,NT_{i,j,t},\;NT_{i,j,t}*\;\;N^{+}$	∀i∈I,j∈J	(25)

The objective function of the model is to minimise the total cost over the planning horizon, which includes two main components: the capital recovery and operating costs of shovels $f_{1}(t)$ and trucks $f_{2}(t)$ . Constraint (12) ensures that the total number of shovels of type *i* operating in the first year is equal to the number of new shovels purchased in the first year. Constraint (13) ensures that the total number of shovels of type *i* operating in subsequent years is the sum of the shovels operating in the previous year and the new shovels purchased in the current year. Constraint (14) ensures that the total number of trucks of type *j* for shovel type *i* operating in the first year is equal to the number of new trucks purchased in the first year. Constraint (15) ensures that the total number of trucks of type *j* for shovel type *i* operating in subsequent years is the sum of the trucks operating in the previous year and the new trucks purchased in the current year. Constraints (16) and (17) maintain a proportional relationship between the number of shovels and trucks. The Big-M method (Hillier and Lieberman, 2010) is employed here as a large constant value that activates or deactivates the constraints, effectively linking the variables in these relationships. Constraint (18) defines the match factor for each year *t*, which is a function of the total number of trucks, the loading time, and the cycle time. Constraints (19) and (20) ensure that the match factor in year *t* remains within the specified minimum and maximum limits, respectively. Constraint (21) ensures that the total production in year *t* is equal to the sum of the production contributions from each truck and shovel combination, considering the number of trips, truck capacity, and availability. Constraint (22) ensures that the total production in year *t* meets the minimum annual production requirement. Constraint (23) ensures that incompatible truck–shovel pairs will not be allocated. Finally, Constraint (24) ensures that the match factor and production are non-negative, while Constraint (25) ensures that the number of new shovels and trucks, as well as the total number of shovels and trucks, are non-negative integers.

#### Heterogeneous equipment selection model with existing equipment

In many real-world operations, fleets consist not only of newly acquired equipment but also of pre-existing assets with varying levels of wear, residual economic life, and operational performance. Some pieces could be secondhand equipment, from previous operations, or leased from third parties. The revised model addresses this by incorporating the characteristics of pre-existing equipment into the decision-making process. These characteristics include different capital recovery, operating cost, and availability structures based on their remaining operational life. Thus, an equipment management strategy can be accomplished until the end of operation life. The additional sets and parameters introduced to effectively represent existing equipment are as follows:

**Table:** 

Set	
L	Set of existing shovel IDs
K	Set of existing truck IDs
Parameter	
$NL_{i,l,t}^{e}$	Number of existing shovel type *i*, ID number *l* in year t
$NT_{i,j,k,t}^{e}$	Number of existing truck type *j* for shovel type *i*, ID number *k* in year t
$C_{i,l,t}^{c,\delta e}$	Capital recovery of existing shovel type *i*, ID number *l* in year t
$C_{\;j,k,t}^{c,\zeta e}$	Capital recovery of existing truck type *j*, ID number *k* in year t
$C_{i,l,t}^{o,\delta e}$	Operating cost of existing shovel type *i*, ID number *l* in year t
$C_{\;j,k,t}^{o,\zeta e}$	Operating cost of existing truck type *j*, ID number *k* in year t
$A_{\;j,k,t}^{e}$	Availability of existing truck type *j*, ID number *k* in year t

The objective function of the model is adjusted to include the capital recovery and operating costs associated with both new and existing equipment, as shown in Eq. (11′):

(11) $$Min\;\underset{t\in T}{\sum }\;f_{1}(t)+f_{2}(t)+f_{3}(t)+f_{4}(t)$$

$$\begin{array}{rl} f_{1}(t)= & \underset{i\in I}{\sum }\;\underset{t_{2}\in T,\;t\geq t_{2}}{\sum }\;(C_{i,(t-t_{2}+1)}^{c,\delta }+C_{i,(t-t_{2}+1)}^{o,\delta })\\ & \cdot NL_{i,t_{2}}*\cdot w\forall \;t\in T \end{array}$$

$$\begin{array}{rl} f_{2}(t)= & \underset{i\in I}{\sum }\;\underset{\;j\in J}{\sum }\;\underset{t_{2}\in T,\;t\geq t_{2}}{\sum }\;(C_{\;j,(t-t_{2}+1)}^{c,\zeta }+C_{\;j,(t-t_{2}+1)}^{o,\zeta })\\ & \cdot NT_{i,j,t_{2}}*\cdot w\forall \;t\in T \end{array}$$

$$f_{3}(t)=\underset{i\in I}{\sum }\;\underset{l\in L}{\sum }\;(C_{i,l,t}^{c,\delta e}+C_{i,l,t}^{o,\delta e})\cdot NL_{i,l,t}^{e}\cdot w\forall \;t\in T$$

$$f_{4}(t)=\underset{i\in I}{\sum }\;\underset{\;j\in J}{\sum }\;\underset{k\in K}{\sum }\;(C_{\;j,k,t}^{c,\zeta e}+C_{\;j,k,t}^{o,\zeta e})\cdot NT_{i,j,k,t}^{e}\cdot w\forall \;t\in T$$

The modified objective function builds on the previous model by adding $f_{3}(t)$ and $f_{4}(t)$ to account for the costs associated with existing equipment. While $f_{1}(t)$ and $f_{2}(t)$ , explained in the previous model, capture the capital recovery and operating costs of new shovels and trucks, $f_{3}(t)$ and $f_{4}(t)$ extend the model to include similar costs for existing shovels and trucks, respectively.

To reflect the dynamic nature of the fleet, the equipment balancing constraints are also modified to account for existing trucks and shovels. These Constraints (12′–15′) now track the total number of operational units in each period as a combination of newly acquired equipment, existing equipment carried forward, and equipment that is retired or phased out due to reaching the end of its operational life:

(12) $$NL_{i,t}=NL_{i,t}*+\underset{l\in L}{\sum }\;NL_{i,l,t}^{e}\forall \;i\in I,\;t=1$$

(13) $$\begin{array}{r} NL_{i,t}=NL_{i,t-1}+NL_{i,t}*+\underset{l\in L}{\sum }\;(NL_{i,l,t}^{e}-NL_{i,l,t-1}^{e})\\ \forall \;i\in I,\;t>1 \end{array}$$

(14) $$NT_{i,j,t}=NT_{i,j,t}*+\underset{k\in K}{\sum }\;NT_{i,j,k,t}^{e}\forall \;i\in I,\;j\in J,\;t=1$$

(15) $$\begin{array}{rl} NT_{i,j,t}= & NT_{i,j,t-1}+NT_{i,j,t}*+\underset{k\in K}{\sum }\;(NT_{i,j,k,t}^{e}-NT_{i,j,k,t-1}^{e})\\ & \forall \;i\in I,j\in J,\;t>1 \end{array}$$

The production constraint is also revised to integrate the contributions of existing equipment alongside new acquisitions, as shown in equation (21′):

(21) $$P_{t}=\underset{i\in I}{\sum }\;\underset{\;j\in J}{\sum }\;\left[NTR_{i,j,t}\cdot L_{j}\left(\underset{t_{2}\in T,\;t\geq t_{2}}{\sum }\;NT_{i,j,t_{2}}*\cdot A_{\;j,(t-t_{2}+1)})+\underset{k\in K}{\sum }\;NT_{i,j,k,t}^{e}\cdot A_{\;j,k,t}^{e}\right)\right]\;\forall \;t\in T$$

The revised model's enhancements provide a more robust framework for fleet optimisation, extending its applicability to scenarios where existing resources play a significant role in operational planning. By considering the full range of available equipment, the model allows for a nuanced analysis of trade-offs between acquiring new assets and utilising existing ones. This capability is crucial for industries where cost minimisation and resource efficiency are primary concerns. The integration of existing equipment dynamics bridges the gap between theoretical optimisation and practical implementation, ensuring that the model is well-suited for real-world applications.

## Case study

**Figure 2.**
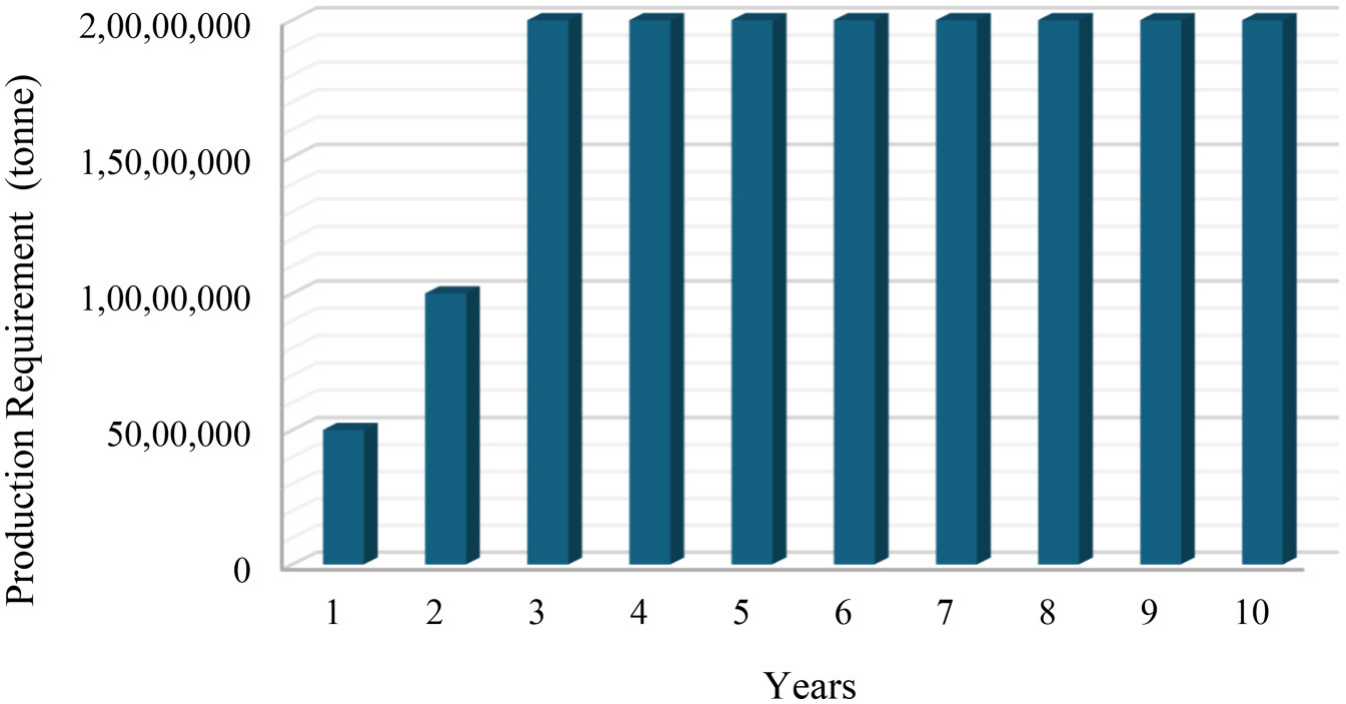
Minimum annual production requirement.

The study employs two distinct scenarios for an open-pit mine project. The first scenario focuses on an open-pit mine project designed as a greenfield project, where no pre-existing infrastructure or equipment is available. In this scenario, the project starts from scratch, requiring the acquisition and deployment of all necessary equipment to commence production. The mine is assumed to operate for a total of 10 years, with production running 20 h per day and 300 days per year, resulting in an annual operational time of 6000 h. The minimum annual production requirements of mine are presented in Figure 2, which illustrates a ramp-up period during the initial years followed by steady-state production.

The ramp-up period is a critical aspect of the greenfield project design. In the first year, the annual minimum production target is set at 5 million tonnes, increasing to 10 million tonnes in the second year, before stabilising at 20 million tonnes from the third year onward. This gradual increase in production allows the mine to overcome initial operational challenges. Additionally, the ramp-up strategy minimises financial risks by allowing incremental capital investments while ensuring that production aligns with infrastructure readiness and market demand. In addition, the model enforces match factor constraints, ensuring that the match factor remains between 0.7 and 1.0, thereby preventing both under- and over-utilisation of the fleet.

The analysis includes three shovel types and six truck types that are commonly utilised in the commercial mining industry. These equipment types were carefully chosen to represent standard industry practices and provide a robust foundation for evaluating optimal combinations under the defined project conditions. According to the equipment specifications provided by the manufacturers, the total operational lifetime of the shovels is 60,000 h, while the trucks have a lifetime of 80,000 h. Considering the 6000 operational hours per year, shovels are assumed to have a lifetime of 10 years, while trucks are assumed to have a lifetime of 13 years. The capacities of the selected shovel and truck types are presented in Table 1.

**Table 1.** Equipment types and capacities.

Truck type	Truck capacity^a^	Shovel type	Shovel capacity^a^
TK73	60	SH8	15
TK75	70	SH12	22
TK77	100	SH16	30
TK85	140		
TK89	200		
TK93	240		

^a^ Capacities are in metric tonnes. In the industry standards, shovel capacities are given in m^3^. However, since the bulk density of rock is known, it is given in tonnes.

Under the scope of this analysis, the number of passes required for each shovel–truck combination is presented in Table 2. This data reflects the compatibility and efficiency of the various pairings, where the numbers indicate the number of shovel swings needed to fully load a truck. The information is derived from equipment specification charts provided by manufacturers, which are widely regarded as standard references in industry. These specifications already account for typical fill and swell factors associated with standard rock conditions.

**Table 2.** Number of passes for each shovel–truck combination.

Equipment type	TK73	TK75	TK77	TK85	TK89	TK93
SH8	4	5	7	-	-	-
SH12	-	3	4–5	6–7	-	-
SH16	-	-	3	4–5	6	7–8

**Figure 3.**
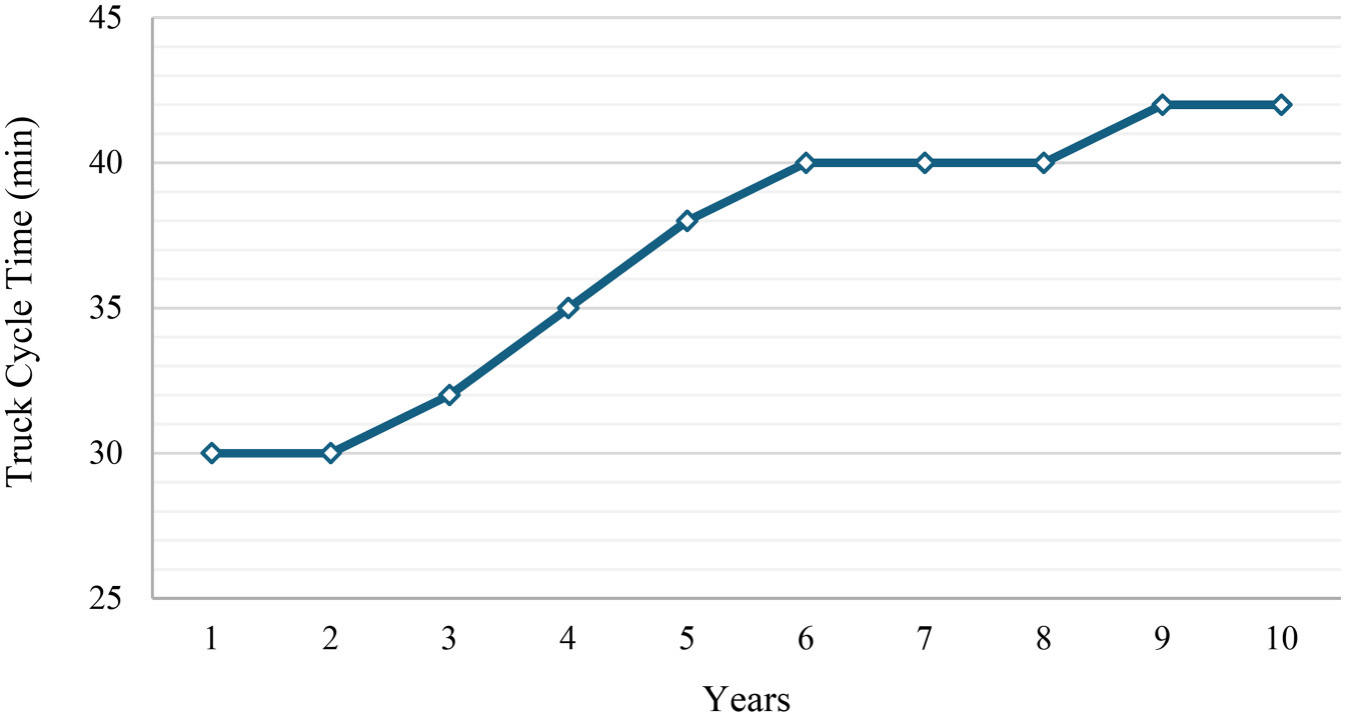
Annual average truck cycle time over 10 years.

Combinations marked with a dash signify that the pairing is either incompatible due to equipment specifications, such as dimensional constraints, or operationally inefficient. These pairings are excluded from the analysis and incorporated as constraints to ensure practical and effective equipment selection. By assuming a single swing time of 40 s, the corresponding loading times for each compatible shovel–truck combination are calculated. Moreover, the average truck cycle times are assumed to remain the same across all truck types. However, they vary by operational year, reflecting the impact of mining advancement on haulage distances and cycle durations. Figure 3 illustrates the variation in annual average truck cycle time over the mine life. Based on these cycle times and the assumption of 6000 operational hours per year, the corresponding total number of trips made by a truck is calculated annually for each year of operation.

**Figure 4.**
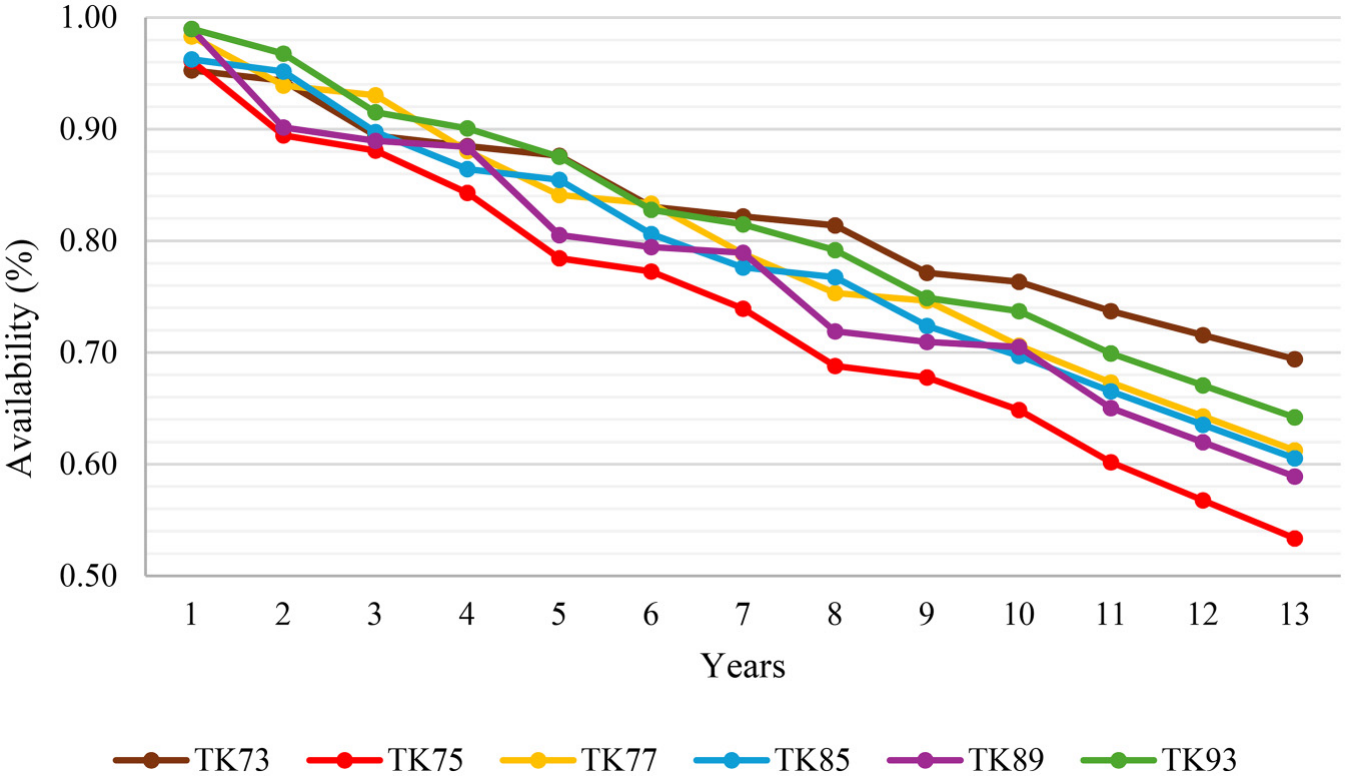
Annual availability of trucks over the operational lifetime.

For the availability calculations, operational data on MTTF and MTTR for each truck type was obtained from historical failure and repair records at an open-pit mining operation. Specifically, MTTF was calculated as the average time interval between consecutive failures for each truck type and MTTR as the average duration required to complete repairs and restore the truck to operational status. These calculations were performed separately for each truck model to reflect operational and maintenance differences across the fleet. Based on this dataset, the instantaneous availability at the start of each operational year was determined for each truck type. In the model, the availability value at the beginning of each year is used as the representative value for that entire year and is assumed constant during the annual period. This approach allows availability to degrade incrementally with equipment age while maintaining consistency with yearly planning intervals. The annual sequence of these availability values over the operational lifetime of the trucks is presented in Figure 4, forming the basis for modelling equipment performance within the analysis.

The cost analysis in this study incorporates both operating and capital costs for the selected equipment. Yearly operating costs for the shovels and trucks were obtained from the Mine and Mill Equipment Costs: An Estimator's Guide (CostMine, 2016). These cost values were first adjusted to the current year using relevant cost indices to account for inflation and changes in economic conditions. Additionally, operating costs were assumed to increase by 3% annually to reflect inflationary trends and rising expenses. The hourly operating cost values for each equipment type, calculated over their operational lifespans of 10 years for shovels and 13 years for trucks, are presented in Table 3.

**Table 3.** Hourly operating costs for shovels and trucks over their respective lifespans ($/hour).

Years	SH8	SH12	SH16	TK73	TK75	TK77	TK85	TK89	TK93
1	208	291	452	69	93	104	164	201	312
2	215	299	466	71	96	108	169	207	321
3	221	308	480	73	99	111	174	213	331
4	228	318	494	75	102	114	180	220	341
5	234	327	509	77	105	118	185	226	351
6	241	337	524	80	108	121	191	233	361
7	249	347	540	82	111	125	196	240	372
8	256	357	556	85	115	128	202	247	383
9	264	368	573	87	118	132	208	255	395
10	272	379	590	90	122	136	214	262	407
11	-	-	-	92	125	140	221	270	419
12	-	-	-	95	129	145	228	278	431
13	-	-	-	98	133	149	234	287	444

Capital cost values for the equipment, also taken from the Mine and Mill Equipment Costs: An Estimator's Guide (CostMine, 2016), were updated to reflect current economic conditions. Using the declining balance method, these capital costs were distributed over the equipment's lifespan to calculate annual capital recovery costs for each equipment type. The declining order of cost values emphasises higher depreciation in the earlier years of equipment use, representing the equipment's diminishing economic value over its lifespan. The hourly capital recovery values for each equipment type are provided in Table 4. This methodology ensures that the financial analysis is consistent with industry-standard accounting practices and incorporates realistic economic assumptions.

**Table 4.** Hourly capital recovery for shovels and trucks over their respective lifespans ($/hour).

Years	SH8	SH12	SH16	TK73	TK75	TK77	TK85	TK89	TK93
1	125	188	321	52	62	77	147	179	221
2	90	137	233	38	45	56	106	129	160
3	65	99	169	27	33	41	77	94	116
4	47	72	122	20	24	29	56	68	84
5	34	52	89	14	17	21	40	49	61
6	25	38	64	10	12	15	29	36	44
7	18	27	47	8	9	11	21	26	32
8	13	20	34	5	7	8	15	19	23
9	9	14	24	4	5	6	11	14	17
10	7	10	18	3	3	4	8	10	12
11	-	-	-	2	2	3	6	7	9
12	-	-	-	2	2	2	4	5	6
13	-	-	-	1	1	2	3	4	5

**Figure 5.**
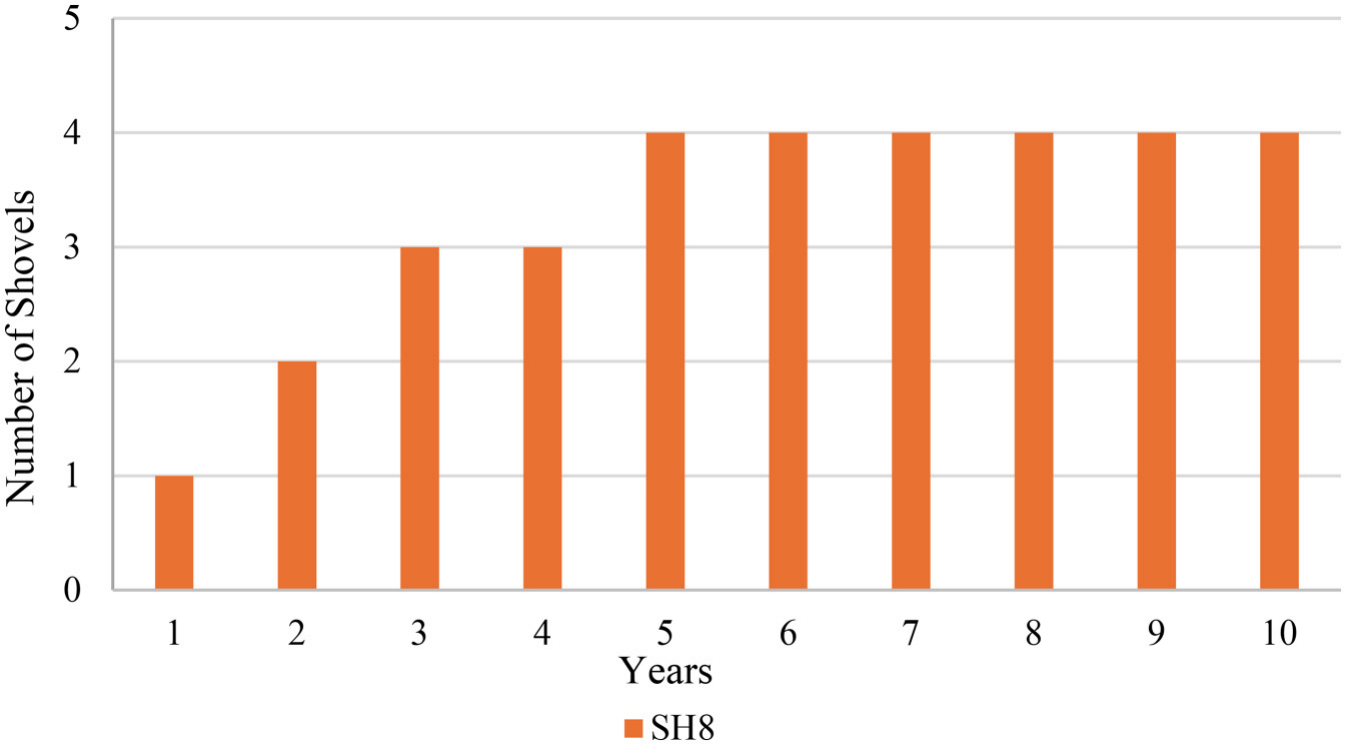
Number of shovels needed in operation over 10 years.

The MINLP model was solved using Python to determine the optimal equipment selection over a 10-year horizon. The results showed that the total cost of the fleet, including capital recovery and operating costs, is $250,080,000. Figure 5 illustrates the acquirement strategy for shovels over the 10-year planning horizon. The results suggest that SH8 shovel type was identified as the most cost-effective and operationally efficient choice under the defined constraints and production scenarios, and therefore, SH12 and SH16 shovel types were not included in the optimal solution.

**Figure 6.**
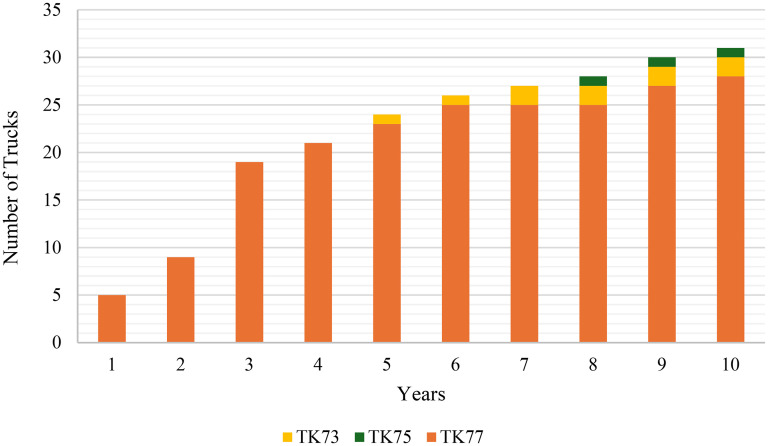
Number of trucks in operation over 10 years.

The figure highlights a gradual increase in the number of SH8 shovel type units during the initial years, reflecting the optimisation model's strategy to scale operations in alignment with production requirements. This phased approach effectively minimises upfront capital expenditure while ensuring that production demands are consistently met throughout the planning period. Similarly, Figure 6 presents the allocation of truck types over 10 years, illustrating the type and number of trucks in operation each year. It indicates that TK77 truck type constitutes the majority of the fleet, with additional contributions from TK73 and TK75 truck types in later years.

TK73 and TK75 truck types are introduced in Years 5 and 8, respectively, to supplement the existing fleet, demonstrating the optimisation model's ability to strategically integrate different equipment types when necessary. Besides, Table 5 demonstrates the achieved match factor and production values by years, as shown below.

**Table 5.** Match factor and production over 10 years.

Year	MF	Production (tonne)
1	0.83	5,880,000
2	0.75	10,344,000
3	0.99	20,486,250
4	1.00	20,035,180
5	0.78	20,300,639
6	0.80	20,316,600
7	0.82	20,046,600
8	0.84	20,046,600
9	0.86	20,051,855
10	0.89	20,117,851

The results in Table 5 indicate that the match factor values across all years consistently fell within the specified range of 0.7 < MF < 1.0, ensuring efficient utilisation of equipment resources. Years 3 and 4 achieved match factors close to 1.0, reflecting peak operational efficiency during these periods. Additionally, the minimum required production targets were met each year throughout the mine life. The slight overachievement in production each year provides operational flexibility, affirming the model's capability to handle long-term planning scenarios effectively.

The second scenario of the case study shifts to a brownfield project, where the mine is already in operation, and some infrastructure and equipment are already in place. Unlike a greenfield project, a brownfield project involves optimising or expanding existing operations to meet new production goals or improve efficiency. This scenario assumes that the mine was initially planned as a 20-year project, and the analysis is conducted at the end of the 10th year of operation. The equipment remaining from the first scenario becomes the initial equipment for the brownfield project. In this context, the goal is to determine the optimal equipment for the remaining 10 years of the mine's life. Table 6 presents the remaining equipment types, their quantities, and their remaining operational lifetimes at the start of the brownfield scenario.

**Table 6.** Remaining equipment and operational hours.

Equipment type	Quantity	Operational hours
SH8	1	6000
	1	12,000
	1	24,000
TK73	1	44,000
	1	56,000
TK75	1	62,000
TK77	5	20,000
	4	26,000
	10	32,000
	2	38,000
	2	44,000
	2	50,000
	2	68,000
	1	74,000

**Figure 7.**
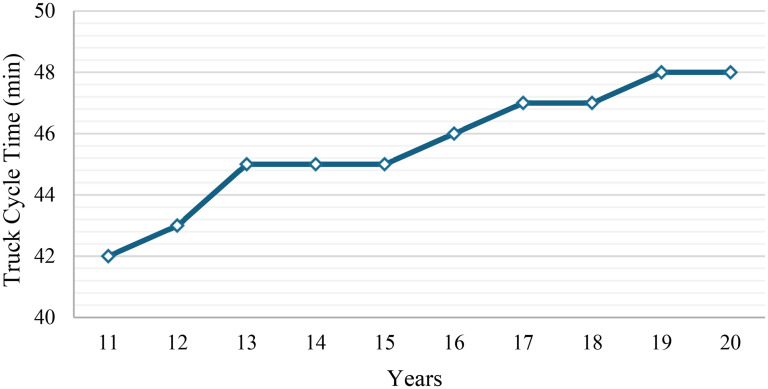
Annual average truck cycle time over the remaining 10 years.

The operational characteristics of the brownfield project remain consistent with those of the greenfield project, including 20 h of daily operation, 300 days per year, and a minimum annual production requirement of 20 million tonnes. Moreover, the same equipment set is used to optimise the acquisition of new equipment, meaning the information presented in Table 1 and Table 2 remains unchanged for the brownfield scenario. For the remaining equipment in the brownfield scenario, the availability, capital recovery, and operating cost values were continued from where they left off at the end of the greenfield scenario. The availability assigned to each piece of pre-existing equipment was based on its cumulative operating years. For example, if a truck had already completed 5 years of operation at the start of the brownfield scenario, its availability in the first year of analysis for the brownfield scenario corresponds to the availability value at Year 6 in the calculated annual availability schedule. This method ensures that the degradation in equipment performance due to aging is accurately represented for both newly acquired and pre-existing assets, with availability values updated annually to reflect the equipment's operational history. Equipment will be removed from the fleet as its operational lifetime expires. This ensures that the analysis accurately reflects the gradual phase-out of existing equipment while allowing for the introduction of new equipment to maintain production targets. Additionally, the average truck cycle time is assumed to change over the remaining mine life due to the advancement of the mine and increasing haul distances. The variation in the annual average truck cycle time for the brownfield scenario is shown in Figure 7.

The MINLP model was modified to reflect a brownfield project and solved using Python to determine the optimal equipment selection over the remaining 10-year horizon. According to the results of the brownfield project, the total cost of the fleet is calculated as $356,826,000. The higher total cost of the brownfield project compared with the greenfield project is primarily due to differences in production strategies and equipment availabilities. In the greenfield project, production ramps up gradually during the first 2 years, leading to lower initial costs. In contrast, the brownfield project operates at full capacity from the start, increasing costs early in the planning horizon. Moreover, the declining availability of older equipment in the brownfield project reduces production efficiency and requires timely replacements to sustain operational targets, contributing to higher overall costs.

**Figure 8.**
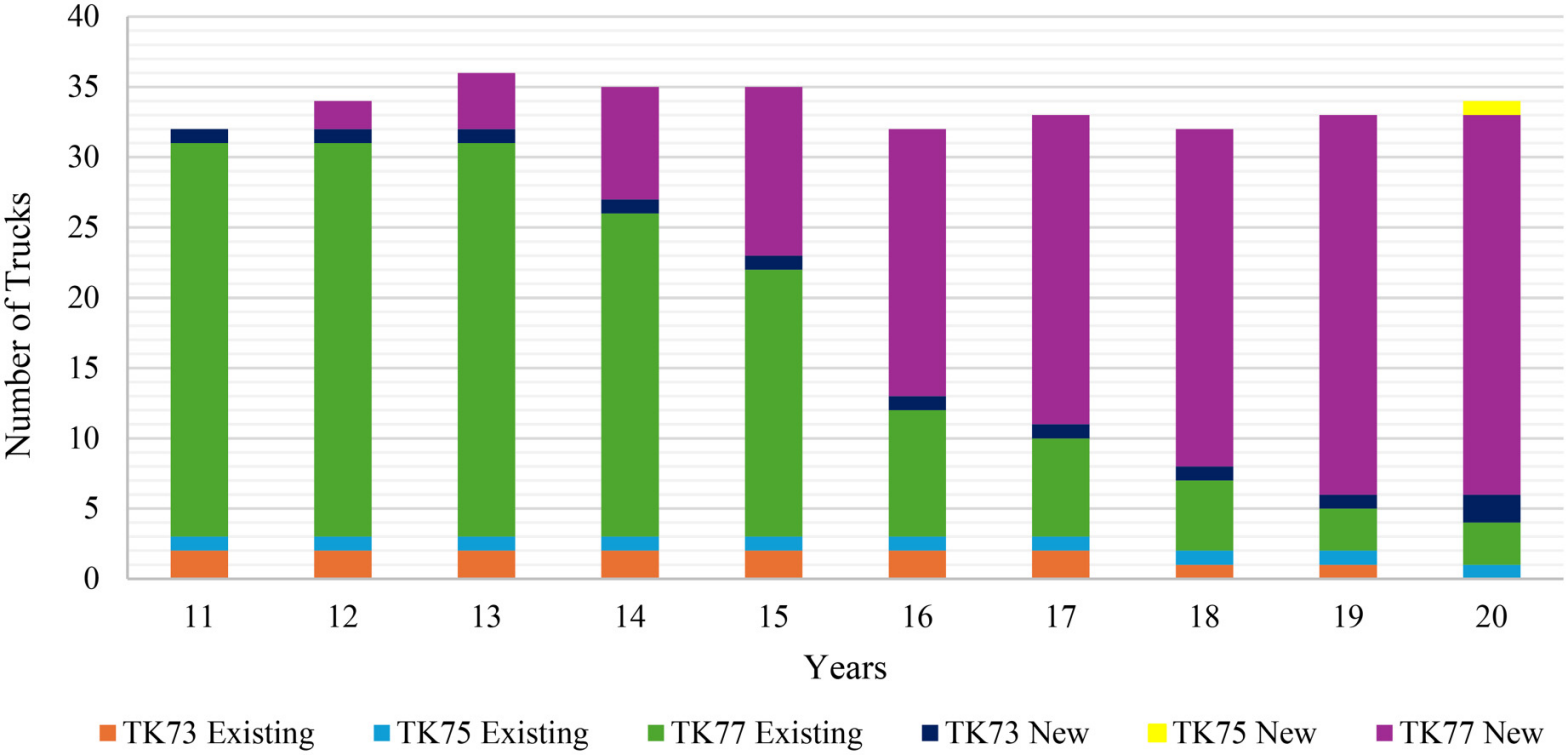
Number of trucks in operation over the remaining 10 years.

Additionally, it is observed that the number of shovels purchased remains identical to the results shown in Figure 5. This is due to the operational life of the shovels, which is also 10 years. As a result, the model consistently replaces each shovel that reaches the end of its operational life with an identical new shovel at the same time intervals observed in the first model. Figure 8 illustrates the number of trucks in operation for the brownfield project over the remaining 10-year planning horizon. It provides a detailed breakdown of the fleet composition by the truck type, highlighting the contributions of both existing and newly acquired equipment. The results in Figure 8 indicate that the existing TK77 truck type forms the foundation of the fleet during the initial years, with additional new TK77 units introduced progressively to maintain production targets as existing trucks reach the end of their operational life. A new unit of the TK73 truck type was also introduced in the first and last year to supplement the fleet, reflecting the model's flexibility in incorporating different truck types to optimise operations. Notably, the TK75 truck type plays a minimal role, with its inclusion appearing to be driven by specific operational needs where its deployment is most effective.

Besides, the results for Years 11–20, as shown in Table 7, indicate that the match factor consistently falls within the required range, with values generally hovering around the upper limit, reflecting a well-balanced truck-to-shovel allocation.

**Table 7.** Match factor and production over the remaining 10 years.

Year	MF	Production (tonne)
11	0.90	20,062,997
12	0.94	20,347,309
13	0.96	20,125,600
14	0.93	20,198,400
15	0.93	20,613,600
16	0.83	20,169,167
17	0.84	20,415,065
18	0.82	20,019,094
19	0.83	20,141,250
20	0.84	20,024,250

The results demonstrate the robustness of the brownfield project model in sustaining high production rates and efficient resource utilisation throughout the 20-year horizon.

## Conclusions

This study presents an in-depth analysis of equipment selection optimisation through the application of a MINLP model. The research develops a robust and adaptable framework that balances operational efficiency and cost minimisation while addressing key considerations such as equipment compatibility, match factors, capital recovery, operating costs, and availability metrics. By incorporating match factor constraints, the model ensures efficient allocation and coordination of resources, minimising idle time and maximising productivity. The comprehensive cost assessment integrates capital recovery and operating costs over multiple periods, using updated cost data and inflation adjustments to provide a realistic economic evaluation. Established accounting practices, such as the declining balance method, further enhance the reliability of cost projections. Additionally, the use of availability metrics derived from real-world operational data ensures an accurate representation of equipment performance and lifecycle dynamics.

By applying the framework to both greenfield and brownfield scenarios, this study offers insights into the different challenges and strategies required for projects at various stages of development. The greenfield scenario emphasises the importance of phased implementation, allowing for a gradual ramp-up in production capacity to minimise initial capital expenditures while aligning with resource availability. This approach facilitates cost-efficient equipment acquisition while maintaining steady progress towards achieving operational targets. In contrast, the brownfield scenario highlights the challenges of integrating existing resources with newly acquired equipment. The model demonstrates the need for adaptive strategies to manage resource availability, accounts for the declining performance of aging assets, and sustains full-capacity operations. By balancing the use of existing equipment with strategic replacements, the optimisation framework ensures that operational efficiency and resource utilisation are maintained. These findings demonstrate the flexibility of the framework in addressing varying operational constraints and project conditions, offering a valuable tool for decision-making across diverse contexts.

While the case study focuses on material handling systems in an industrial setting, the broader implications of this research extend to any sector that relies on efficient resource allocation and fleet management. The dynamic, multi-period optimisation framework addresses key gaps in traditional static, single-period analyses, providing a practical tool for long-term planning and operational efficiency. Its ability to integrate advanced decision-making techniques with real-world constraints makes it broadly applicable to industries such as logistics, manufacturing, and construction. By offering a structured and data-driven approach to resource optimisation, the framework has the potential to improve cost efficiency, reduce downtime, and enhance overall system performance in a variety of applications. While the current model provides a robust foundation for equipment selection and fleet optimisation, certain site-specific working conditions—such as ground material variations, haul road gradients, tire life, detailed haul road conditions or the effects of carryback—were not explicitly modelled and were instead assumed to be consistent or to have a minimal impact within the planning horizon. This assumption supports model tractability and broad applicability, though consideration of these operational variabilities in future studies could further enhance the model's precision for site-specific applications.

Future research could also build upon this foundation by incorporating environmental considerations, such as energy consumption and emissions, into the optimisation framework to align with sustainability goals. Additionally, evaluating the impacts of emerging technologies, such as electric-powered vehicle, automation, and digitalisation, on resource allocation and fleet optimisation could provide new perspectives. Expanding the framework to include other material handling systems or integrating real-time data for dynamic, real-time decision-making could further enhance its utility.

## References

[bibr1-25726668251348712] Akulker H Aydin E (2023) Optimal design and operation of a multi-energy microgrid using mixed-integer nonlinear programming: Impact of carbon cap and trade system and taxing on equipment selections. Applied Energy 330: 120313.

[bibr2-25726668251348712] Baykasoǧlu A Kaplanoglu V Durmuşoglu ZDU , et al. (2013) Integrating fuzzy DEMATEL and fuzzy hierarchical TOPSIS methods for truck selection. Expert Systems with Applications 40(3): 899–907.

[bibr3-25726668251348712] Bodziony P Kasztelewicz Z Sawicki P (2016) The problem of multiple criteria selection of the surface mining haul trucks. Archives of Mining Sciences 61(2): 223–243.

[bibr4-25726668251348712] Burt CN Caccetta L (2007) Match factor for heterogeneous truck and loader fleets. International Journal of Mining, Reclamation and Environment 21(4): 262–270.

[bibr5-25726668251348712] Burt CN Caccetta L (2008) Erratum: Match factor for heterogeneous truck and loader fleets (international journal of mining, reclamation and environment (2007) vol. 21 (4) (262-270)). International Journal of Mining, Reclamation and Environment 22(1): 84–85.

[bibr6-25726668251348712] Burt CN Caccetta L Hill S , et al. (2005) Models for mining equipment selection. MODSIM05: International Congress on Modelling and Simulation: Advances and Applications for Management and Decision Making, Proceedings: 1730–1736.

[bibr7-25726668251348712] Čelebić M Bajić D Bajić S , et al. (2024) Development of an Integrated Model for Open-Pit-Mine Discontinuous Haulage System Optimization. Sustainability (Switzerland) 16(8): 3156. 10.3390/su16083156.

[bibr8-25726668251348712] Chakraborty S Prasad K (2016) A QFD-based expert system for industrial truck selection in manufacturing organizations. Journal of Manufacturing Technology Management 27(6): 800–817.

[bibr9-25726668251348712] Chaowasakoo P Seppälä H Koivo H , et al. (2017) Improving fleet management in mines: The benefit of heterogeneous match factor. European Journal of Operational Research 261(3): 1052–1065.

[bibr10-25726668251348712] CostMine (2016) *Mine and Mill Equipment Costs: An Estimator’s Guide* .

[bibr11-25726668251348712] Dindarloo SR Osanloo M Frimpong S (2015) A stochastic simulation framework for truck and shovel selection and sizing in open pit mines. Journal of the Southern African Institute of Mining and Metallurgy 115(3): 209–219.

[bibr12-25726668251348712] Duran A Praščević N (2021) Allocation and selection of equipment for concrete works using fuzzy linear programming. Building Materials and Structures 64: 195–199.

[bibr13-25726668251348712] Fu Z Topal E Erten O (2014) Optimisation of a mixed truck fleet schedule through a mathematical model considering a new truck-purchase option. Transactions of the Institutions of Mining and Metallurgy, Section A: Mining Technology 123(1): 30–35.

[bibr14-25726668251348712] Goswami SS Behera DK (2021) Solving material handling equipment selection problems in an industry with the help of entropy integrated COPRAS and ARAS MCDM techniques. Process Integration and Optimization for Sustainability 5(4): 947–973.

[bibr15-25726668251348712] Hazrathosseini A Moradi Afrapoli A (2024) Transition to intelligent fleet management systems in open pit mines: A critical review on application of reinforcement-learning-based systems. Mining Technology: Transactions of the Institutions of Mining and Metallurgy 133(1): 50–73.

[bibr16-25726668251348712] Hillier FS Lieberman GJ (2010). Introduction to Operations Research (9th ed.). New York, NY: McGraw-Hill.

[bibr17-25726668251348712] Horňáková N Jurík L Hrablik Chovanová H , et al. (2021) AHP Method application in selection of appropriate material handling equipment in selected industrial enterprise. Wireless Networks 27(3): 1683–1691.

[bibr18-25726668251348712] Huayanca D Bujaico G Delgado A (2023) Application of Discrete-Event Simulation for Truck Fleet Estimation at an Open-Pit Copper Mine in Peru. Applied Sciences (Switzerland) 13(7): 4093. 10.3390/app13074093.

[bibr19-25726668251348712] Krause A Musingwini C (2007) Modelling open pit shovel-truck systems using the machine repair model. Journal of the Southern African Institute of Mining and Metallurgy 107(8): 469–476.

[bibr20-25726668251348712] Leland Blank PE Anthony Tarquin PE (2012) Engineering Economy (7th ed.). New York, NY: McGraw-Hill.

[bibr21-25726668251348712] Malli T Mizrak Ozfirat P Yetkin ME , et al. (2021) Truck selection with the fuzzy-wsm method in transportation systems of open pit mines. Tehnicki Vjesnik 28(1): 58–64.

[bibr22-25726668251348712] Mohtasham M Mirzaei-Nasirabad H Askari-Nasab H , et al. (2021) Truck fleet size selection in open-pit mines based on the match factor using a MINLP model. Mining Technology: Transactions of the Institute of Mining and Metallurgy 130(3): 159–175.

[bibr23-25726668251348712] Mohtasham M Mirzaei-Nasirabad H Askari-Nasab H , et al. (2022) Multi-stage optimization framework for the real-time truck decision problem in open-pit mines: A case study on sungun copper mine. International Journal of Mining, Reclamation and Environment 36(7): 461–491.

[bibr24-25726668251348712] Moradi-Afrapoli A Tabesh M Askari-Nasab H (2019) A multiple objective transportation problem approach to dynamic truck dispatching in surface mines. European Journal of Operational Research 276(1): 331–342. https://doi.org/10.1016/j.ejor.2019.01.008.

[bibr25-25726668251348712] Mustaffa NK (2022) Alternative configurations of earthmoving loading practices toward emissions reduction. Journal of Construction Engineering and Management 148(1): 04021177.

[bibr26-25726668251348712] Nguyen HT Md Dawal SZ Nukman Y , et al. (2016) An integrated MCDM model for conveyor equipment evaluation and selection in an FMC based on a fuzzy AHP and fuzzy ARAS in the presence of vagueness. PLoS ONE 11(4): 1–26. 10.1371/journal.pone.0153222 PMC4829176 27070543

[bibr27-25726668251348712] Ortiz CEA Curi A Campos PH (2014) The use ofsimulation in fleet selection and equipment sizing in mining. In: Mine Planning and Equipment Selection. Cham, Switzerland: Springer, 869–877.

[bibr28-25726668251348712] Oukil A (2024) Selecting material handling equipment through a market weight scheme based DEA cross-efficiency approach. International Journal of Management Science and Engineering Management 19(1): 1–14.

[bibr29-25726668251348712] Patyk M Bodziony P (2018) Analysis of multiple criteria selection and application of APEKS method in haul truck mining transport process. E3S Web of Conferences 71. 10.1051/e3sconf/20187100003.

[bibr30-25726668251348712] Patyk M Bodziony P (2022) Application of the analytical hierarchy process to select the most appropriate mining equipment for the exploitation of secondary deposits. Energies 15(16): 5979. 10.3390/en15165979.

[bibr31-25726668251348712] Pham H (2003) Handbook of Reliability Engineering (Hoang Pham, Ed.). London, UK: Springer-Verlag, 117–138.

[bibr32-25726668251348712] Rakhmangulov A Burmistrov K Osintsev N (2024) Multi-criteria system’s design methodology for selecting open pits dump trucks. Sustainability 16(2): 63.

[bibr33-25726668251348712] Shehadeh A Alshboul O Tatari O , et al. (2022) Selection of heavy machinery for earthwork activities: A multi-objective optimization approach using a genetic algorithm. Alexandria Engineering Journal 61(10): 7555–7569.

[bibr34-25726668251348712] Tan Y Undram C Miwa K , et al. (2012) Operations modeling and analysis of open pit copper mining using GPS tracking data. Proceedings of the 2012 Winter Simulation Conference: 1–12. https://doi.org/10.1109/WSC.2012.6465062.

[bibr35-25726668251348712] Tiile R Kaba F Aouad N , et al. (2016) Shovel-Truck haulage analysis using stochastic discrete event simulation. International Journal of Science and Research (IJSR) 5(11): 495–500.

[bibr36-25726668251348712] Tobias PA Trindade D (2011). Repairable Systems Part I: Nonparametric Analysis and Renewal Processes. In Applied Reliability (3rd Edition). Philadelphia, PA: CRC Press, 417–470. 10.1201/b11787-17

[bibr37-25726668251348712] Ulutaş A (2020) Stacker selection with PSI and WEDBA methods. International Journal of Contemporary Economics and Administrative Sciences 10(2): 493–504.

[bibr38-25726668251348712] Ulutaş A Topal A Karabasevic D , et al. (2023) Selection of a forklift for a cargo company with fuzzy BWM and fuzzy MCRAT methods. Axioms 12(5): 467. 10.3390/axioms12050467.

[bibr39-25726668251348712] Yang J Liu F Dong Y , et al. (2022) Multiple-objective optimization of a reconfigurable assembly system via equipment selection and sequence planning. Computers & Industrial Engineering 172: 108519.

[bibr40-25726668251348712] Yavuz M (2016) Equipment selection by using fuzzy TOPSIS method. IOP Conference Series: Earth and Environmental Science 44(4): 042040. 10.1088/1755-1315/44/4/042040.

[bibr41-25726668251348712] Yazdani-Chamzini A (2014) An integrated fuzzy multi criteria group decision making model for handling equipment selection. Journal of Civil Engineering and Management 20(5): 660–673.

[bibr42-25726668251348712] Zaerpour N Volbeda R Gharehgozli A (2019) Automated or manual storage systems: Do throughput and storage capacity matter? Information Systems and Operational Research 57(1): 99–120.

[bibr43-25726668251348712] Zhao Q. Zhang X. Wang P. (2024). Multi-t ype equipment selection and quantity decision optimization in intelligent warehouse. IEEE Access, 12, 63515–63527.

